# Long term immunologic consequences of experimental stroke and mucosal tolerance

**DOI:** 10.1186/2040-7378-1-3

**Published:** 2009-10-21

**Authors:** J Michael Gee, Dannielle Zierath, Jessica Hadwin, Anna Savos, Angela Kalil, Matthew Thullbery, Kyra J Becker

**Affiliations:** 1 Department of Neurology, University of Washington School of Medicine Seattle Washington, USA

## Abstract

**Background:**

An inflammatory insult following middle cerebral artery occlusion (MCAO) is associated with a predisposition to develop a deleterious autoimmune response to the brain antigen myelin basic protein (MBP). Induction of immunologic tolerance to brain antigens prior to MCAO prevents this deleterious autoimmune response and is associated with better functional outcome early after stroke. In this study, we sought to determine the long term immunologic consequences of experimental stroke and induction of mucosal tolerance.

**Methods:**

Male Lewis rats were tolerized to MBP or ovalbumin (OVA) by intranasal administration prior to MCAO and administration of lipopolysaccharide (LPS). Neurological outcome was assessed at set points after MCAO and animals sacrificed at 3 months; the immune response to MBP in brain and spleen was determined using ELISPOT assay and degree of cellular inflammatory brain infiltrate assessed by immunocytochemistry.

**Results:**

Animals that developed a pro-inflammatory (TH1) response to MBP experienced worse outcome, while those that developed a regulatory response (TREG) experienced better outcome. A TREG response in spleen was also associated with decreased inflammation and an increase in the number of FoxP3 positive cells in brain. In this study, tolerization to MBP prior to MCAO was associated with a tendency to develop a TH1 response to MBP by 3 months after MCAO.

**Conclusion:**

These data show that induction of immunological tolerance to MBP is associated with improved outcome after stroke. This study, however, raises concern about the potential for inadvertent induction of detrimental autoimmunity through mucosal administration of antigen.

## Introduction

There is a complex interplay between the central nervous system (CNS) and the systemic immune system; the immune response appears to contribute to ischemic brain injury and cerebral ischemia affects the systemic immune response. Immediately after experimental stroke, peripheral blood lymphocytes and splenocytes become activated and are capable of secreting massive amounts of pro-inflammatory cytokines [1]. In concert with this systemic response, there is inflammation within the injured brain, and strategies that limit the inflammatory response within the brain improve outcome from experimental stroke [2]. Modulation of the immune response following stroke, however, has yet to translate into clinical benefit.

Despite the initial inflammatory response in the brain and periphery after stroke, the immune system later becomes incapable of adequately responding to pathogens, predisposing animals to infection [3]. One potential benefit of this "systemic immunodepression" is that it limits the chance of developing an autoimmune response to the brain antigens exposed to the immune system by brain injury [4]. In fact, we previously showed that if an immune response to CNS antigens occurs following experimental stroke, it is usually that of a regulatory response (TREG) [5]. By inducing systemic inflammation in the peri-infarct period, however, there appears to be a fundamental change in how the immune system responds to the CNS antigens exposed in injured brain; a detrimental autoimmune response emerges, and this autoimmune response is associated with worse functional outcome 1 month after middle cerebral artery occlusion (MCAO) [5,6]. This observation may help explain why patients who experience infection in the immediate post-stroke period have increased morbidity and mortality. Using the paradigm of mucosal tolerance, however, we demonstrated that induction of a TREG response to the brain antigen myelin basic protein (MBP) prior to cerebral ischemia could prevent development of the deleterious autoimmune response to this antigen and improve outcome (as assessed 1 month after MCAO)[6] There are, however, documented concerns about the long term consequences of mucosal tolerance/immune deviation therapy [7-9]. In the current experiments, we extended the period of follow up to 3 months after MCAO to assess for adverse outcomes; immunocytochemistry (ICC) was also performed to assess the degree of cellular inflammation in the brain.

## Materials and methods

### Animals

Experiments were approved by the Institution's Animal Care and Use Committee. Male Lewis rats (250 to 300 g) were used for all studies. Rats were handled prior to tests/surgical procedures and housed 3 per cage to eliminate differences in socialization.

### Tolerization Schedule

**Figure 1:**
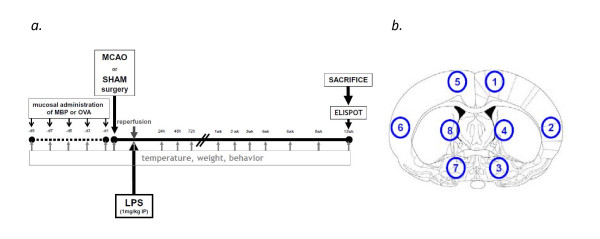
**Experimental paradigm (*a*)**. Areas of interest for quantifying inflammatory cellular infiltrates into brain (***b***).

The experimental paradigm is outlined in Figure 1a. Prior to sham surgery or stroke, either bovine MBP (100 μg/40 μl; N = 30) or ovalbumin (OVA; 100 μg/40 μl; N = 29) was instilled into each nostril every other day for a total of 5 doses; surgery was performed 1-2 days after the last instillation. (Heterologous MBP has been repeated shown to induce immunologic tolerance to MBP which is "clinically" meaningful [10].)

### Middle Cerebral Artery Occlusion (MCAO)

Anesthesia was induced with 5% and maintained with 1.5% isoflurane. After midline neck incision, the right common carotid, internal carotid and pterygopalantine arteries were ligated. A monofilament suture (4.0) was inserted into the common carotid artery and advanced into the internal carotid artery [11]; 20 OVA tolerized and 25 MBP tolerized animals were subjected to MCAO. Animals were maintained at normothermia during surgery. Reperfusion was performed 3 hours after MCAO. In sham-operated animals (9 OVA tolerized and 5 MBP tolerized), the suture was inserted into the carotid but not advanced. Rectal temperature and body weight were assessed at set time intervals. Animals were sacrificed 3 months after MCAO or sham-surgery. (Data from a previous study in which animals were sacrificed at 1 month after MCAO or sham-surgery are also included for reference [6].)

### Lipopolysaccharide Administration

All animals received LPS (serotype 026:B6; 1 mg/kg intraperitoneally) 3 hours after MCAO (at reperfusion) or sham surgery.

### Neurological Outcome

Neurological status was assessed at set time points after MCAO; tests included a modification of the Bederson scale and an adaptation of the 'sticky tape test' [12,13]. Sensorimotor performance was assessed using a rotating drum or "rotarod"; average time to fall (100 secs maximum) from a rod rotating at 5 rpms over three trials was recorded. Data for non-surviving animals are included to the time of their death.

### ELISPOT Assays

Animals for ELISPOT were sacrificed 3 months after MCAO/sham surgery and mononuclear cells (MNCs) isolated from the brain and spleen using previously described methods [5,14]. MNCs were cultured (1 × 10^5 ^cells/well) for 48 hours in 96-well plates (MultiScreen^®^-IP; Millipore) in media alone or in media supplemented with MBP (25 μg/mL) or OVA (25 μg/mL). All experiments were performed in triplicate. Antigen specific secretion of IFN-γ (in comparison to unstimulated cells) was used as an indicator of the TH1 response; antigen specific secretion of TGF-β1 was used as an indicator of the TREG response. Spots were counted under a dissecting microscope by two independent investigators blinded to treatment status. The ratio of the increase in the number of MBP or OVA-specific cells secreting IFN-γ to that of the increase in MBP or OVA-specific cells secreting TGF-β1 was determined. Based on previous data from our laboratory, the lower interquartile range for this ratio in animals sensitized to MBP (by injection in complete Freund's adjuvant) was 1.48 [15]. We thus considered animals to have a TH1(+) response to MBP if the ratio of the IFN:TGF response was ≥ 1.48; conversely, a TREG was considered to be induced if this ratio was ≤ 0.68 (*ie*. 1.00 ÷ 1.48).

The number of ELISPOT assays performed on MNCs from brain was less than that on spleen given that some brains were frozen for ICC (N = 8 for OVA tolerized and N = 17 for MBP tolerized animals subjected to MCAO and N = 6 for OVA tolerized and N = 3 for MBP tolerized animals subjected to sham-surgery).

### Enzyme-Linked ImmunoSorbent Assays (ELISAs)

Blood was collected by cardiac puncture at the time of sacrifice. Serum was stored at -80° until use. Fractalkine levels were assessed using a commercially available kit (R&D Systems). Titers of IgG antibodies specific for MBP were measured using indirect ELISA; data are presented as relative absorption units.

### Immunocytochemistry

Brains were snap frozen in isopentane and sections (20 μm) stored at -80°C until use. The following monoclonal antibodies were used: clone W3/25 (AbD Serotec) directed against CD4 in T cells, clone OX-8 (AbD Serotec) directed against CD8 in T cells, clone OX-33 to detect CD45 on B cells (GenWay), clone #10/78 directed against CD161 on natural killer (NK) cells (Serotec), and clone # FJK-16s directed against Foxp3 (eBioscience). Sections were fixed in acetone and blocked with Superblock buffer (Thermo Scientific); ICC was performed using the immunoperoxidase method and sections were counterstained with cresyl violet. The numbers of positively stained cells were quantified on coronal brain slices corresponding to the center of the infarct (bregma -0.26 mm). Cells were counted in 6 different high power (100×) fields at 4 different locations in both the infarcted (1-4) and non-infarcted hemisphere (5-8); Figure 1b depicts these areas. Hemispheric volume was determined using NIH image and expressed as the ratio between the ischemic and non-ischemic hemisphere.

### Statistics

Categorical data were evaluated using the χ^2^-test statistic. Parametric data were compared using the *t*-test, paired *t*-test or analysis of variance (ANOVA) and are expressed as mean ± standard error of the mean (SEM). Non-parametric data were compared using the Mann-Whitney U-test. Significance was set at P ≤ 0.05.

## Results

### MBP versus OVA tolerized Animals

**Figure 2:**
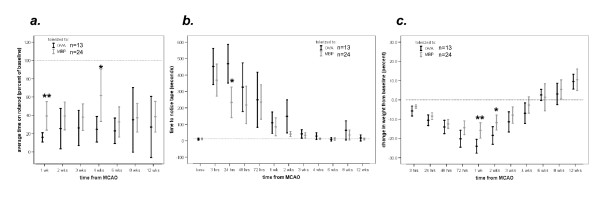
**MBP tolerized animals perform better on the rotarod (*a*), demonstrate quicker recognition of adhesive tape on the paw (*b*) and gain weight more rapidly (*c*) than OVA tolerized animals up to 1 month after MCAO; the benefits are no longer apparent at 3 months**. Point estimates represent the mean and the bars represent the standard error of the mean (*P < 0.05, **P < 0.01 using the *t*-test).

Following MCAO, mortality was higher in OVA (7/20) than MBP (1/25) tolerized animals (*P *= 0.007). All deaths occurred within 24 hours of MCAO except for 1 OVA tolerized animal that died between months 2 and 3. Among surviving animals, the temperatures did not differ at any point after stroke (data not shown). There was a general trend towards MBP tolerized animals to regain weight faster than OVA tolerized animals and to perform better on tests of functional outcome, but these differences were not sustained (Figure 2).

There were more splenocytes in MBP than OVA tolerized animals (13.8 × 10^8 ^± 3.1 × 10^8 ^*vs*. 5.5 × 10^8 ^± 1.1 × 10^8^; *P *= 0.018) at sacrifice; the numbers of brain monocytes in MBP and OVA tolerized animals were similar. The concentrations of circulating fractalkine and MBP antibodies were also similar in MBP and OVA tolerized animals undergoing MCAO.

Among sham-operated animals, 1/5 MBP and 0/9 OVA tolerized animals died (NS). There were no differences in the temperatures, neurological scores or performance on behavioral tests (sticky tape test and rotarod) of MBP and OVA tolerized sham-operated animals at any time point after surgery. The concentrations of circulating fractalkine and MBP antibodies at the time of sacrifice did not differ between sham-operated MBP tolerized and sham-operated OVA tolerized animals.

Sham-operated animals performed markedly better on all behavioral outcome measures than their counterparts undergoing MCAO. Three months after surgery, OVA tolerized animals undergoing MCAO had higher titers of MBP antibodies than OVA tolerized animals undergoing sham surgery (0.345 ± 0.053 *vs*. 0.187 ± 0.021 relative units; *P *= 0.015); antibody titers to MBP were similar among ischemic and sham-operated MBP tolerized animals.

### Cellular responses in Sham-Operated Animals

Among sham-operated animals, mucosal administration of OVA was associated with a significantly attenuated TH1 response to OVA in spleen at 1 month in comparison to non-OVA tolerized animals (0.67 ± 0.06 *vs*. 1.20 ± 0.17; *P *= 0.004); at 3 months, these differences were no longer apparent. The immune response to OVA in brain did not differ between OVA tolerized and non-OVA tolerized sham-operated animals. Similarly, animals tolerized to MBP had an attenuated TH1 response to MBP in spleen at 1 month after sham surgery (0.70 ± 0.12 *vs*. 1.03 ± 0.09; *P *= 0.05); at 3 months, these differences were no longer apparent. The immune response to MBP in brain did not differ between MBP tolerized and OVA tolerized sham-operated animals. In both OVA tolerized and MBP tolerized animals, there was an increase in the TH1 response to the respective antigens in spleen from 1 month to 3 months; 0.67 ± 0.06 *vs*. 1.70 ± 0.17; P = 0.001 for OVA and 0.70 ± 0.12 *vs*. 1.12 ± 0.12; *P *= 0.07 for MBP.

### TH1 responses following MCAO

**Figure 3:**
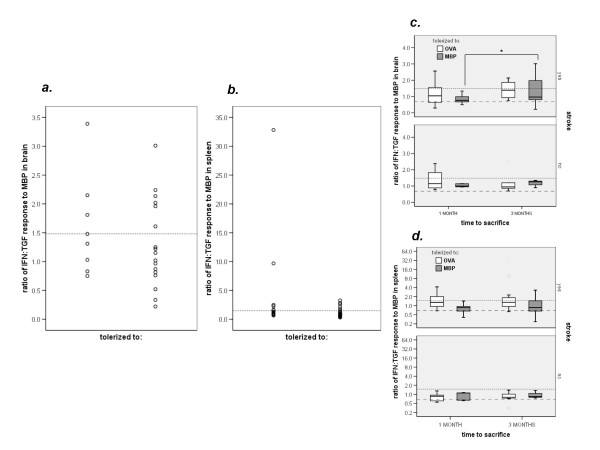
**Individual animal data showing the number of MBP specific IFN and TGF secreting cells in brain (*a*) and spleen (*b*) among OVA and MBP tolerized animals (note the difference in the scales on the Y-axis)**. The magnitude of the TH1 response to MBP among MBP tolerized animals was greater at 3 months than 1 month in brain (***c***) but not spleen (***d***).

The cellular immune responses to OVA in both brain and spleen were similar in OVA tolerized and non-OVA tolerized animals following MCAO at 1 month and 3 months following MCAO; immune responses to OVA were not predictive of outcome at either time point. We previously showed that induction of mucosal tolerance to MBP prior to MCAO and LPS administration prevented the post-ischemic TH1 response to MBP at 1 month [6]. In the current study where animals were examined 3 months after MCAO, however, no differences were seen between MBP and OVA tolerized animals in either the *proportion *of animals developing a TH1(+) response to MBP or the *magnitude *of that response (Table 1 and Figure 3). (Data from our 1 month study are also displayed in Table 1 and Figure 3; comparison of these data show that the proportion of MBP tolerized animals that developed a TH1(+) response to MBP in brain is higher at 3 months than at 1 month.)

**Table 1:** Immune status at 1 and 3 months following middle cerebral artery occlusion.

*time to sacrifice*	*1 month*			*3 months*			*1 vs. 3 months*	
***brain***	***OVA*** ***N = 17***	***MBP*** ***N = 16***	***P***	***OVA*** ***N = 8***	***MBP*** ***N = 17***	***P***	***OVA***	***MBP***

*TH1*	*6 (35)*	*1 (6)*	*0.033*	*4 (50)*	*6 (35)*	*NS*	*NS*	*0.041*

*TREG*	*5 (29)*	*5 (31)*	*NS*	*0*	*3 (18)*	*NS*	*0.086*	*NS*

***spleen***	***OVA*** ***N = 21***	***MBP*** ***N = 21***	***P***	***OVA*** ***N = 13***	***MBP*** ***N = 22***	***P***	***OVA***	***MBP***

*TH1*	*8 (38)*	*1 (6)*	*0.001*	*6 (46)*	*5 (23)*	*NS*	*NS*	*0.089*

*TREG*	*0*	*7 (33)*	*0.001*	*1 (8)*	*6 (27)*	*NS*	*NS*	*NS*

Animals are considered to have a TH1(+) response to MBP if the ratio of the relative increase in the number of MBP-specific IFN secreting cells to the relative increase in the number of MBP-specific TGF secreting cells is at least 1.48. Conversely, they are considered to have a TREG response to MBP if the ***ratio ***of the relative increase in the number of MBP-specific IFN secreting cells to the relative increase in the number of MBP-specific TGF secreting cells is ≤ 1 ÷ 1.48 (0.68).

**Figure 4:**
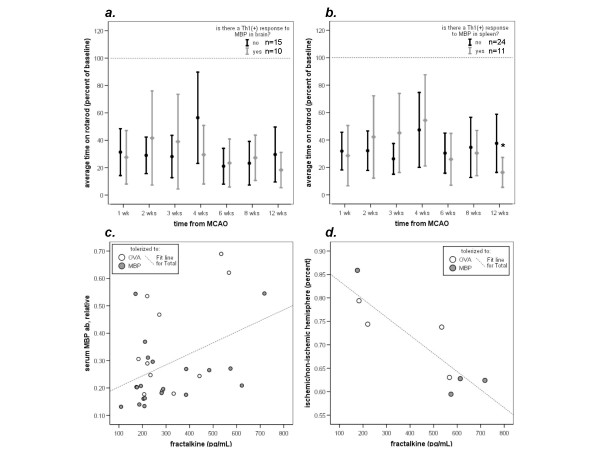
**The TH1 response to MBP in brain is not predictive of rotarod performance (*a*), but animals with a TH1 response to MBP in spleen have a shorter latency to fall from the rotarod (*ie*. worse performance) 3 months after MCAO than animals without a TH1 response to MBP in spleen (*b*)**. Point estimates represent the mean and the bars represent the standard error of the mean (*P < 0.05 using the *t*-test). There is a correlation between the humoral response to MBP (antibody titer) and the concentration of circulating fractalkine (***c***) and the concentration of circulating fractalkine is correlated with the degree of brain atrophy (***d***) (*ie*. higher concentrations of fractalkine are associated with more brain atrophy).

There was no difference in performance on the rotarod among animals with or without a TH1(+) response to MBP in brain (Figure 4a). Animals with a TH1(+) response to MBP in spleen, however, performed worse on the rotarod at 3 months after stroke onset (Figure 4b). Among animals considered to have a TH1(+) response to MBP in brain, there was a strong correlation between the degree of that response and the time it took to attend to a piece of sticky tape on the paw (r = 0.872; *P *= 0.001). For animals with a TH1(+) response to MBP in spleen, there was a similar correlation between the degree of TH1(+) response to MBP in spleen and performance on the sticky tape test (r = 0.965; *P *< 0.001).

Our previous data suggested that circulating levels of fractalkine might be a useful marker of an ongoing immune response in brain at 1 month after MCAO[6] At 3 months, however, there were no differences in the concentrations of circulating fractalkine among animals with a TH1(+) response to MBP (in either spleen or brain) and those without. Higher concentrations of fractalkine were, however, associated with higher titers of anti-MBP antibodies (r = 0.413, *P *= 0.021) (Figure 4c) and increased brain atrophy (r = 0.879, *P *= 0.004) (Figure 4d). There was also a modest correlation between the cellular immune response to MBP and the humoral response to MBP (r = 0.395, *P *= 0.028).

### TREG Response to MBP following MCAO

**Figure 5:**
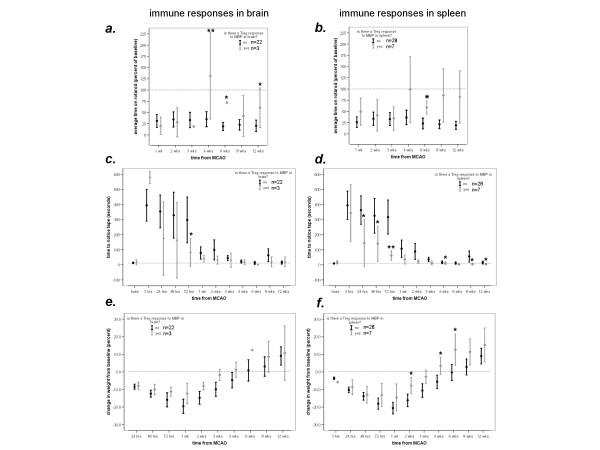
**Animals with a demonstrated TREG response to MBP perform better on the rotarod (*a*, *b*), have shorter latency to react to adhesive tape on the paw (*c*, *d*) and regain weight faster (*e*, *f*) than animals without a TREG response**. (*P < 0.05, **P < 0.01 using the *t*-test).

At one month after MCAO, animals tolerized to MBP were more likely to evidence a TREG response to MBP than animals tolerized to OVA [6]. In the current study, however, we found that a TREG response to MBP was difficult to detect 3 months after MCAO. Only 6/22 (27%) MBP tolerized (and 1/13 [8%] OVA tolerized) animals evidenced a TREG response to MBP in spleen; the number of animals with a TREG response to MBP in brain was even smaller (3/17 [18%] MBP tolerized and 0/8 OVA tolerized animals) (Table 1). As shown in Figure 5, animals with a TREG response to MBP in either brain or spleen tended to have longer latencies to fall from the rotarod (Figure 5a, b), react more quickly to a stimulus on their paw (Figure 5c, d) and regain weight more quickly (Figure 5e, f) than animals without a TREG response.

### Inflammatory Infiltrates in Brain

**Figure 6:**
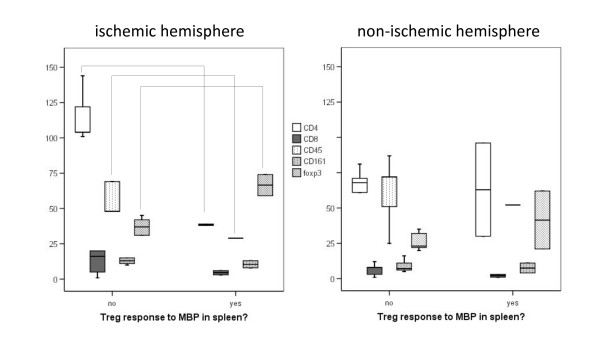
**There are fewer CD4^+^, fewer CD45^+ ^and more Foxp3^+ ^cells in the ischemic hemisphere of the brain in animals with a TREG response to MBP in spleen at 3 months**. (*P < 0.05 using the Mann-Whitney U test).

In a subgroup of animals, ICC was performed on brain. There were no differences in the number of CD4^+^, CD8^+^, CD45^+^, CD161^+^, or Foxp3^+ ^cells in the ischemic hemisphere (regions 1-4; Figure 1b) of MBP tolerized and non-tolerized animals (data not shown); MBP tolerized animals, however, had fewer CD4^+ ^cells in the non-ischemic hemisphere of (*P *= 0.036). Animals with a TREG response to MBP in spleen (N = 2) had fewer CD4^+^, fewer CD45^+ ^and more Foxp3^+ ^cells in the infarcted hemisphere than those without a TREG response (N = 6); in the non-ischemic hemisphere, the numbers of these cells were similar in animals with and without a TREG response (Figure 6). The numbers of CD8^+ ^and CD161^+ ^cells did not differ between animals with and without a TREG response to MBP.

## Discussion

Previous studies demonstrated that mucosal administration of CNS or vascular antigens in experimental stroke was associated with decreased infarct volume and improved outcome in the hours to days after stroke onset [14,16-19]. We recently showed that the benefits of inducing mucosal tolerance to brain antigens (*ie*. MBP) extend to at least 1 month after MCAO [6]. In the current study, however, we find that this benefit does not extend to 3 months after MCAO. Similar to findings in our previous study, MBP tolerized animals evidenced improved performance on the rotarod and appeared to regain weight more quickly than OVA tolerized animals up to 1 month after MCAO, but this benefit was not sustained past the 1 month mark (Figure 2). One possible explanation for this disparity is that induction of mucosal tolerance to MBP only hastens recovery from stroke, and that OVA tolerized animals "catch up" over time. Another possible explanation for the loss of benefit after 1 month is that the initial treatment (mucosal administration of MBP) leads to a delayed worsening of function. Indeed, if one compares the performance of MBP tolerized animals on the rotarod at 4 weeks after MCAO to that at later time points (Figure 2a), it suggests that there is a deterioration in function after 1 month.

The paradigm for induction of tolerance in this study was successful in attenuating the TH1 response to the antigen administered, as demonstrated by the immune responses in sham-operated animals at 1 month after surgery. By 3 months after sham-surgery, however, the effect of the tolerizing regimen could no longer be detected (in fact, there was an increase in the TH1 response to the antigen to which the animal was "tolerized"). Following MCAO, we detected a higher proportion of MBP tolerized animals with a TH1(+) response to MBP at 3 months than at 1 month. This finding raises the possibility that mucosal administration of (auto) antigens could induce detrimental autoimmune responses. It is important to note that the animals in these studies received LPS at the time of MCAO; it is unclear whether the same tendency toward at TH1(+) response to MBP would be seen in MBP tolerized animals that had not been treated with LPS.

Based on experimental data, there are compelling reasons to pursue mucosal tolerance as a therapy for patients with a variety of autoimmune diseases. The results of clinical studies to date, however, have been disappointing [20,21]. While there are many potential explanations for the failure of mucosal tolerance in clinical trials, there is concern that mucosal administration of antigen can, at least in some instances, lead to detrimental autoimmunity. For instance, mucosal administration of antigen during experimental autoimmune encephalomyelitis (EAE) may exacerbate inflammation and oral administration of high dose insulin may induce autoimmune diabetes [7,8]. In addition, it is known that mucosal administration of antigen can induce antibody production (a Th2 response) to the administered antigen [22,23]. Such humoral responses may also be detrimental, as demonstrated in a non-human primate model of EAE where therapy with soluble myelin oligodendrocyte glycoprotein (MOG) was associated with the late development of pathogenic MOG antibodies [9]. A major and important difference between most laboratory studies and clinical trials is the duration of follow up. In the laboratory setting, animals are generally followed for only a short duration of time; in the clinical arena, patients are generally followed for a much longer period of time and there is more opportunity for toxicities to emerge.

In this study we considered MBP specific induction of IFN-γ as the marker for the TH1 response and MBP specific induction of TGFβ-1 as the marker for the inducible TREG response; animals that evidenced a TH1 response to MBP had worse outcome from stroke while those that evidenced a TREG response to MBP had better outcome. There is now a robust literature suggesting that TH17 cells are the key effector cells in many autoimmune diseases [24]. Further, the ratio of antigen specific TH17 to TH1 cells can determine the nature of the autoimmune disease [25]. Future studies will need to address the role of TH17 cells in our stroke model. As mentioned, inducible TREG cells are characterized by antigen specific secretion of TGFβ-1 [21,26]; inducible TREG cells also secret IL-10 [27]. Irrespective of how the TREG cells are identified, animals that evidence a TREG response to MBP appear to have a better outcome from MCAO [6,14,16-19]. These data further support the fact that the immune response to CNS antigens affects outcome from experimental stroke.

Given the apparent importance of a TREG response to MBP for good outcome from MCAO, a viable therapeutic strategy would be one that is associated with an enhanced and sustained increase in the TREG response to defined brain antigens. That we were unable to demonstrate a significant difference in the proportion of MBP and OVA tolerized animals with a TREG response to MBP at 3 months may relate to the possibility that MBP treated animals "deviated" towards a TH1 response by 3 months, as already discussed. While functional TREG cells can be found within a few days of mucosal antigen administration [28], T cell function begins to normalize after several weeks [29]. The loss of the TREG response to both OVA and MBP among sham-operated OVA tolerized and MBP tolerized animals, respective, at 3 months is consistent with this "normalization of function". Studies have shown that "booster" doses of antigen can be used to sustain the beneficial effects of mucosal tolerance [18,30,31]. In concert, these findings suggest that induction of CNS specific TREG cells after stroke may be an effective stroke therapy, but this induction must be done in a manner that leads to a sustained population of these regulatory cells and does not carry a risk of precipitating subsequent deleterious autoimmunity.

## Competing interests

The authors declare that they have no competing interests.

## Authors' contributions

All authors have read and approved the final manuscript.

JMG and MT performed the majority of the experiments detailed in this manuscript.

DZ, JH and AS assisted in these experiments and performed behavioral testing as well as immunocytochemistry (and its quantification).

AK is the senior researcher in the laboratory and provided assistance with performance of ELISPOT and ELISA assays.

KJB conceived of the experiments, obtained funding, interpreted the data and wrote the manuscript.
